# The FKBP51 Glucocorticoid Receptor Co-Chaperone: Regulation, Function, and Implications in Health and Disease

**DOI:** 10.3390/ijms18122614

**Published:** 2017-12-05

**Authors:** Gabriel R. Fries, Nils C. Gassen, Theo Rein

**Affiliations:** 1 Translational Psychiatry Program, Department of Psychiatry and Behavioral Sciences, McGovern Medical School, The University of Texas Health Science Center at Houston, Houston, TX 77054, USA; 2 Department of Translational Science in Psychiatry, Max Planck Institute of Psychiatry, 80804 Munich, Germany; ncgassen@psych.mpg.de

**Keywords:** chaperone, glucocorticoid receptor, FKBP51, FKBP5, signaling pathway, drug

## Abstract

Among the chaperones and co-chaperones regulating the glucocorticoid receptor (GR), FK506 binding protein (FKBP) 51 is the most intensely investigated across different disciplines. This review provides an update on the role of the different co-chaperones of Hsp70 and Hsp90 in the regulation of GR function. The development leading to the focus on FKBP51 is outlined. Further, a survey of the vast literature on the mechanism and function of FKBP51 is provided. This includes its structure and biochemical function, its regulation on different levels—transcription, post-transcription, and post-translation—and its function in signaling pathways. The evidence portraying FKBP51 as a scaffolding protein organizing protein complexes rather than a chaperone contributing to the folding of individual proteins is collated. Finally, FKBP51’s involvement in physiology and disease is outlined, and the promising efforts in developing drugs targeting FKBP51 are discussed.

## 1. Introduction

**Figure 1:**
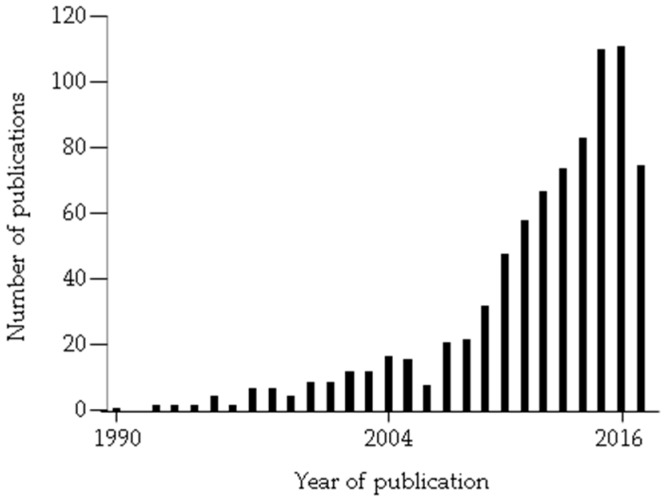
Number of publications on FKBP51 per year since 1990. The numbers are based on a Pubmed search that included FKBP5, FKBP51, FKBP54, FKBP 5, FKBP 51, and FKBP 54. The initial publications described FKBP51 as immunophilin p54. For 2017, citations were retrieved October 30 of this year.

The original discovery of FK506 binding protein (FKBP) 51 was described more than 25 years ago alongside the analysis of steroid receptor complex components [1]. Accordingly, steroid receptors (SRs) and the glucocorticoid receptor (GR) in particular served as the main experimental systems for the initial investigations of FKBP51’s function and mechanism [2,3,4,5], while additional functions of FKBP51 emerged soon afterward [6]. Scientists in several laboratories characterized FKBP51 as a strong inhibitor of GR function [7,8,9,10]. Since GR is a crucial part of the stress hormone axis, also known as HPA (hypothalamus–pituitary–adrenals) axis, and since increasing evidence became available for dysregulated GR in stress-related diseases, such as depression [11], the finding of FKBP51 as an inhibitor of GR inspired a genotype association study on depression [12]. This study discovered that polymorphisms of the FKBP51-encoding gene *FKBP5* were associated with stress hormone axis reactivity and response to antidepressants [12] and amplified interest in FKBP51 research worldwide (see Figure 1 for a summary of the number of FKBP51-publications per year since 1990), along with the discovery of several additional functions of FKBP51 [6].

Given the importance of GR in the initial characterization of FKBP51, we will first provide an update of the contribution of chaperones and co-chaperones to GR function (Section 2), with a focus on FKBP51 and a brief introduction into its structure and biochemical function (Section 3). This is followed by a survey of the literature on regulation and function of FKBP51 (Section 4 and Section 5) and on drug development efforts (Section 6) and by concluding remarks (Section 7).

## 2. Glucocorticoid Receptor Co-Chaperones, Focus on FKBP51

The glucocorticoid receptor (GR) belongs to the large superfamily of nuclear receptors (NRs), which typically act as transcriptional regulators in a ligand-dependent manner [13,14,15]. They share a common structure that became apparent already before any of their genomic sequence was known [16]: an N-terminal activator domain, a central DNA binding domain, and a C-terminal activator domain 2, which is also the domain of ligand binding [17]. NRs are grouped into seven subfamilies, NR0-6, based on their homology [18]. GR, designated as NR3C1, belongs to the NR3 subfamily, which encompasses all steroid hormone receptors.

GR is functionally involved in a broad spectrum of physiological processes, including the immune, cardiovascular, reproductive, nervous, and metabolic system [19]. This vast range of physiological functions requires intricate control of its activity [20]. In addition to molecular chaperones and co-chaperones, which predominantly are known as regulators of folding and conformation of GR in the cytosol, there is a vast array of cofactors that determine GR’s activity as a transcription factor on the chromatin. These are not the subject of this survey, excellent reviews have been published [20,21,22].

### 2.1. Complexity of GR Regulating Chaperones and Co-Chaperones

The first confirmed chaperone found associated with SRs was heat shock protein (Hsp) 90 [4,23,24]. Hsp90 is a highly abundant protein in eukaryotic cells making up 1–2% of the cytosolic protein content under non-stress conditions and up to 5% under stress conditions [25], now known as a key folding and assembly platform involved in numerous cellular processes [26,27,28]. The discovery of the core components of the GR-chaperone heterocomplex and their step-wise, ATP-dependent assembly has been described in several reviews [2,4,5]. Briefly, in vitro reconstitution experiments revealed that five chaperones and co-chaperones suffice to fold GR to a conformation with high affinity hormone binding competence: Hsp40, Hsp70, hop, Hsp90, and p23 [2,29]. The basic maturation step is thought to involve the initial recognition of GR by Hsp40, which leads to association with ATP-bound Hsp70 [30,31,32]. In the next step, Hsp70-Hsp90 organizing protein (hop) promotes the transfer of the partially folded GR to the Hsp90-based folding platform; hop leaves the folding complex at later stages. Hsp90 primarily acts on the hormone binding domain and keeps it in a hormone binding competent state. P23 is a cofactor of Hsp90 that keeps Hsp90 in the ATP-bound state, thus promoting receptor binding of Hsp90 [33]. More recently, the coordinated action of the Hsp70- and Hsp90-based cycles in the folding and maturation of GR has been analyzed in more detail [34].

While the studies in vitro as well as in yeast provided important mechanistic insight into the basic process of steroid receptor folding such as GR [2,4,35], the chaperone action on SRs in their homologous environment in mammalian cells is more complex. Both Hsp70 and Hsp90 are fine-tuned by a vast array of co-chaperones [26,36,37,38,39]. In addition, the functional outcome of chaperone and co-chaperone action also depends on the specific context. For example, p23 appears to have several functions as it is known to act as an Hsp90 co-chaperone, as an autonomous molecular chaperone, and as a prostaglandin E2 synthase [40,41,42,43,44]. With respect to its influence on GR activity, in vivo experiments in mammalian cells provided evidence for both stimulation and inhibition of GR-dependent transcription, possibly relating to experimental conditions including cellular context and expression levels of p23 [45,46,47].

### 2.2. Hsp70 and Hsp90 Cofactors

Of the cofactors of Hsp70 and Hsp90, only a small fraction has been analyzed for their potential effect on GR. To provide a glimpse of the complexity, there are 13 known Hsp70 genes in the human genome [48,49], and a genome-wide analysis has revealed the existence of 41 Hsp40 family members. Of these 41, 34 feature typical J domains that confer association with Hsp70, while 7 have partially conserved J-like domains [50,51]. Hsp70 cofactors with known effects on GR are listed in Table 1, along with a short description and references. More recently added was Hsp70 binding protein 1 (HSPBP1), which, like Bcl2-associated athanogene 1 (BAG1), is a nucleotide exchange factor of Hsp70 [52,53]. Similar to the longer BAG1 isoforms BAG1M and BAG1L [54,55,56,57], increased expression of HSPBP1 reduced GR-dependent transcriptional activity [58].

Most of the known GR-regulating Hsp90 cofactors have been reviewed before [2,3,34,88]. Briefly, an important group of Hsp90 cofactors competes for access to Hsp90 and the associated hetero-complexes by binding to the N-terminal EEVD-motif of Hsp90 through their tetratricopeptide repeat (TPR) motif domain [5,71,89]. This combinatorial assembly creates a wide variety of compositions and regulatory possibilities. Some of the TPR-proteins bring additional biochemical functions to the Hsp90 complex, such as phosphatase activity in the case of PP5 or peptidylprolylisomerase (PPIase) activity in the case of most TPR-containing immunophilins. While clear evidence has been provided for the involvement of PP5’s phosphatase activity in GR function through changing both Hsp90 and GR phosphorylation [79,80,90], the role, if any, of the PPIase activity of GR-regulating immunophilins such as FKBP51 and FKBP52 remains unknown [6,74]. The PPIase domain, however, likely plays a crucial role in regulating GR, with effects on hormone binding affinity and nuclear translocation [7,9,74,75]. The competitive interaction of the TPR proteins with Hsp90 is an example to illustrate the dependency of the influence of a given TPR protein of the cellular context, as its effect is modulated by the abundance and nature of the other available TPR proteins. For example, while FKBP52 potentiates GR function in the yeast model [9,74], its overexpression in mammalian cells produces very little, if any, effect on GR, likely due to the presence of numerous other TPR cofactors that already promote GR function [7,71].

More recently described GR regulating TPR-containing Hsp90 cofactors are CNS1, FKBPL, SGTA, SGT1, Tah1, and XAP2 (Table 1). Both inhibitory and stimulatory effects on GR function have been described for these proteins, depending on their abundance as well as the cellular context [27,39,68,69,76,83,87]. Whether or not they are also involved in the regulation of intranuclear GR mobility and recycling, similarly to other chaperones and cochaperones, remains to be elucidated [45,91,92,93,94,95].

## 3. Structure and Biochemical Function of FKBP51

**Figure 2:**
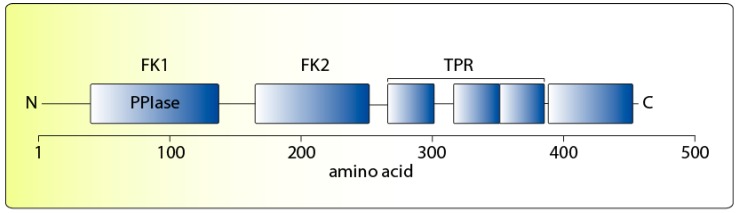
Schematic representation of the FKBP51 protein domain structure. TPR = tetratricopeptide repeat domain conveying Hsp90 interaction.

FKBP51 and FKBP52 share high homology (60%, similarity 70%) and the same domain structure [96] (see also Figure 2). They encompass two N-terminal domains with homology to FKBP12 (FK1 and FK2); only FK1 binds the immunosuppressive drug FK506 (also called tacrolimus) and is biochemically active in expediting the isomerization of peptidyl-prolyl bonds of model peptides with similar activity between FKBP51 and FKBP52 [97,98]. The C-terminal TPR domain endows the two FKBPs with the ability to interact with the EEVD motif at the C-terminal region of Hsp90 [7,89,99], in competition with all the other available TPR proteins in the cell [71,100]. Amino acids downstream of the TPR domain at the very C-terminal end of FKBP51 have also been reported to contribute to Hsp90 binding [101].

The FK1 domain is the primary determinant for the divergent actions of FKBP51 and FKBP52 on GR [7,9]. However, while this PPIase domain is important, its enzymatic function is not. It has been proposed that the association of the FKBPs with Hsp90 in its “closed” conformation [26] enables the FK1 domain to contact the ligand binding domain of GR and other SRs, thereby influencing the conformation and hormone binding affinity [96]. The structures of the FK1s of the two immunophilins differ around the PPIase pocket by several residues of a proline-rich region [74,98,102], which likely causes differential protein interaction and the differential effects on GR activity [74,96]. With respect to GR function, it has not been resolved yet whether FKBP51 contributes a specific effect in inhibiting the receptor or merely competes with GR-stimulating TPR proteins such as FKBP52 out of the Hsp90-based heterocomplex.

FKBP51 has originally been characterized as chaperone and Hsp90 co-chaperone, i.e., involved in the process of folding individual proteins [97,103,104]. As outlined before, it is particularly challenging to prove the in vivo relevance of the PPIase activity and this has not yet been achieved, neither for FKBP51 nor for FKBP52 [6,74]. An increasing number of studies suggests that the most important molecular action of FKBP51 is its ability to serve as protein scaffolder that associates with several regulatory proteins, thus influencing a variety of signal transduction pathways (see Section 5 below for more details).

## 4. Regulation of FKBP51

**Figure 3:**
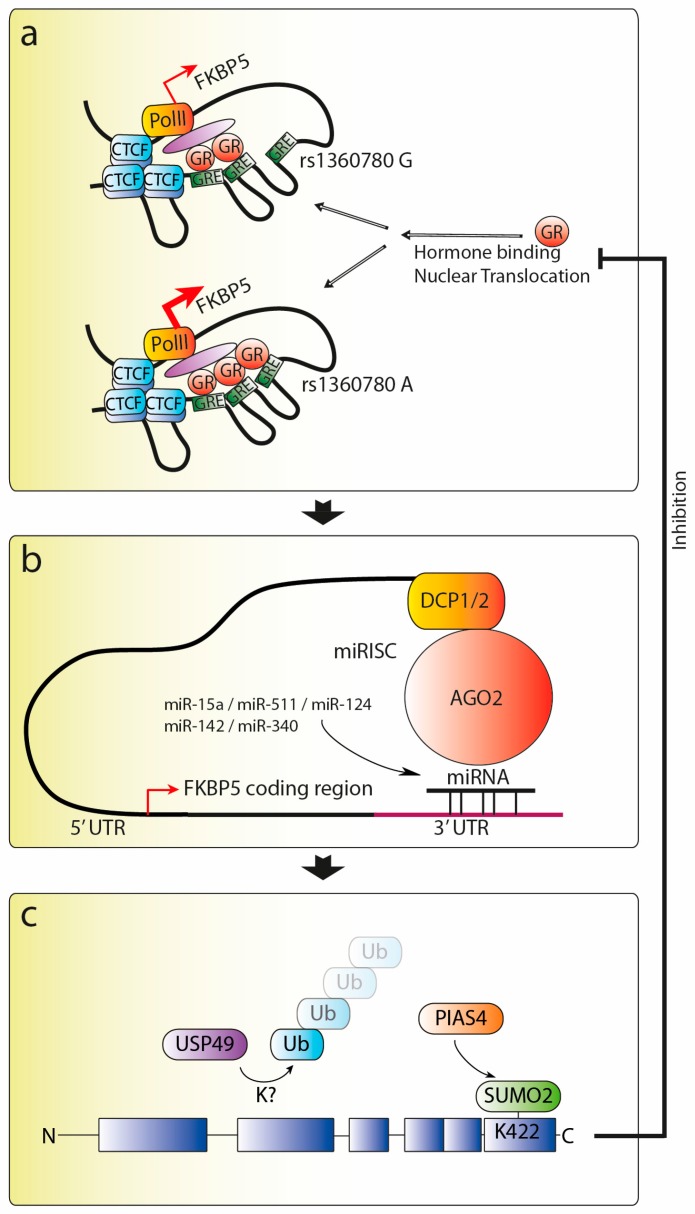
Model of FKBP51’s regulation at different levels. (**a**) Transcriptionally, *FKBP5* is modulated by several single nucleotide polymorphisms, including the functional rs1360780 located within intron 2 and chromatin organizing proteins such as the CCCTC-binding factor. The scheme in Panel A is a hypothetical combination of two chromatin architecture studies [105,106]; (**b**) post-transcriptionally, microRNAs can interfere with the stability and translation of the FKBP5 mRNA molecules; (**c**) post-translationally, FKBP51 is regulated by post-translational modifications, such as ubiquitination (probably at lysines 352 und 376, www.nextprot.org/entry/NX_Q13451/proteomics) and SUMOylation. FKBP51 not only is engaged in various signaling pathways (see below), but also forms an ultrashort negative feedback loop by inhibiting its own production at the transcriptional level. Abbreviations: AGO2 = argonaute 2 (the catalytic subunit of the RISC complex); GR = glucocorticoid receptor; GRE = glucocorticoid response element; CTCF = CCCTC-binding factor; DCP = decapping protein; miRNA = microRNA; PIAS = protein inhibitor of activated STAT; PolII = RNA polymerase II; RISC = RNA-induced silencing complex; SUMO = small ubiquitin-like modifier USP = ubiquitin-specific peptidase; UTR = untranslated region.

Not only is FKBP51 a multifunctional protein (see Section 5), but it is also regulated at multiple layers. Specifically, it can be transcriptionally regulated by several factors, genetic polymorphisms, and DNA methylation, ultimately determining the expression levels of the *FKBP5* gene (see Section 4.1). A couple of microRNAs (miRNAs) have been recently proposed to target the *FKBP5* mRNA, thereby regulating FKBP5’s mRNA stability and/or translation into a functional protein (see Section 4.2). A final layer of modulation takes place post-translationally, influencing the protein stability, and, as we will discuss below, the interaction between FKBP51 and the GR (see Section 4.3). A schematic representation of the different layers of FKBP51 regulation is provided in Figure 3. Altogether, this multitude of regulatory mechanisms allows for a complex modulation of the FKBP51-GR system in response to environmental stimuli and underlies its association with several pathological conditions, as discussed in the following sections.

### 4.1. Transcriptional Regulation

The gene encoding FKBP51 is *FKBP5*, which in humans is located at 6p21.31 and consists of 13 exons and 12 introns spanning more than 150 kb of genomic DNA [107]. The most important hallmark of the *FKBP5* gene, at least with respect to GR regulation, is the presence of functional glucocorticoid responsive elements (GREs) constituting an intracellular short feedback loop [6,106,108]. It has been suggested that *FKBP4* (the gene encoding FKBP52) and *FKBP5* evolved from the same gene through duplication [109]. Nevertheless, glucocorticoid-dependent transcription has been described only for *FKBP5*. The structure of the *FKBP5* gene is very similar to that of *FKBP4*, including their exon–intron boundaries, with the exception that *FKBP5* presents three extra non-coding exons at its 3′ region [107]. Several polymorphisms have been identified in the *FKBP5* gene, including functional ones with known implications in *FKBP5* expression and function [110]. Around 613 single nucleotide polymorphisms (SNPs) and 57 indels (insertions/deletions), including a 3.3 kb deletion, have been discovered so far [107]. One SNP in particular, rs1360780, has been associated with significantly higher FKBP51 levels, as it is linked to differences in GR sensitivity [12]. This same SNP, which is in close proximity to a glucocorticoid-responsive element (GRE) in intron 2 of the *FKBP5* gene, has also been shown to alter the extent of mRNA and protein induction following GR activation [105] (Figure 3a).

As previously mentioned, *FKBP5* is a target for several transcription factors, among which the GR is the most commonly discussed in the context of stress. Chromatin-immunoprecipitations and reporter gene analyses identified GREs located several kilobases upstream and downstream of the transcriptional start site as determinants of the glucocorticoid response of the gene [106,108]. This entails long-range interactions and the involvement of chromatin organizing proteins such as CCCTC-binding factor (CTCF), a general determinant of chromatin domains [105,106,111] (Figure 3b). Interestingly, binding sites for several transcription factors are not primarily located in the promoter region; in fact, several relevant binding sites are located within intronic and distal regions of the *FKBP5* gene [107]. These regions appear to have an even stronger glucocorticoid responsiveness compared to the promoter region itself, with some studies showing an absence or a very low response of the promoter sequence to glucocorticoids [108,112]. Importantly, some of these distal regions show promiscuous responsiveness to other hormone receptors in addition to GR, such as the androgen receptor (AR) and the progesterone receptor (PR) [113]. In particular, AR has been shown to selectively recruit cAMP response element-binding protein to a distal enhancer located 65 kb downstream of the transcription start site in the fifth intron of the *FKBP5* gene, ultimately leading to chromatin remodeling and modulation of its expression [113].

As another means of regulation, binding of transcription and chromatin architecture factors is influenced by DNA methylation at the *FKBP5* locus, which can directly mediate the effects of environmental stimuli on the gene. In mice, chronic (4 weeks) exposure with corticosterone decreases DNA methylation at distinct CpGs along with enhanced expression of *Fkbp5* [114]. In this model, DNA methylation was reversible and reappeared after the cessation of corticosterone exposure. Several studies have provided evidence that DNA methylation across different regions of the *FKBP5* gene can influence its expression and interact with specific polymorphisms. For instance, hypermethylation of the *FKBP5* gene has been associated with a reduced expression of specific transcript variants in patients with MDD [115]. Indirect evidence for a role of DNA methylation in *FKBP5* transcription is often provided by associations with physiological parameters. For example, a positive correlation between *FKBP5* methylation with a thickness of the transverse frontopolar gyrus has been reported in MDD patients [116]. A landmark study has shown that childhood trauma can reduce DNA methylation specifically in the intron 7 of the *FKBP5* gene in subjects presenting the risk allele of the rs1360780 SNP [105], suggesting a complex interplay between genotype, DNA methylation, and the effects of environment on the *FKBP5* gene transcription. DNA methylation apparently also contributes to the upregulation of *FKBP5* transcription with age [117].

Finally, the *FKBP5* transcription has also been shown to be controlled in a tissue-specific manner, probably through a combination of the aforementioned main mechanisms. In particular, a study performed in mice showed that *Fkbp5* is expressed throughout the entire brain, with some regions showing higher expression than others [109]. In particular, regions with a high baseline expression of *Fkbp5*, such as the hippocampus, the premammillary nucleus, and the motor nuclei of the *nervus trigeminus* and *nervus facialis*, tend to show a more modest induction of *Fkbp5* expression in response to dexamethasone treatment (a synthetic glucocorticoid analog) [109]. In other words, dexamethasone treatment can induce a much more prominent induction of *Fkbp5* expression in tissues where its baseline expression is typically low, such as the hypothalamic paraventricular nucleus or the central amygdala, in line with the reported role of FKBP51 as a negative regulator of GR sensitivity [109].

### 4.2. Post-Transcriptional Regulation

Different studies have suggested a post-transcriptional regulation of *FKBP5* expression by means of modulating its mRNA stability. Independent studies indicate the role of specific miRNAs through interference with *FKBP5* mRNA stability and translation by binding to the 3’UTR region. After the processing and biogenesis of mature miRNAs, these regulatory molecules are incorporated into a ribonuclear particle to form the RNA-induced gene silencing complex (RISC) as part of their translational repressor role [118]. An efficient search complex for specific target mRNAs is also facilitated by the association of miRNAs with argonaute (Ago) proteins [119]. Not only has *FKBP5* mRNA been detected in the argonaute RISC catalytic component 2 (Ago2) complex [120], but it has also been shown to bind argonaute and the trinucleotide repeat-containing proteins, which are both part of miRNA-induced silencing complexes [107].

So far, five different miRNA molecules have been reported or predicted to bind to the *FKBP5* mRNA: miR-124 [107], miR-15a [120], miR-142 [120], miR-340 [120], and miR-511 [121] (Figure 3b). Specifically, miR-124 was shown to bind to *FKBP5*’s exon 9 using data from photoactivatable ribonucleoside-enhanced crosslinking and immunoprecipitation (PAR-CLIP) [107], although this finding has yet to be replicated. Additionally, by investigating the effects of chronic stress in mice, a recent study found that an increase in miR-15a levels in the amygdala-Ago2 complex was associated with a concomitant reduction in the levels of *FKBP5* mRNA and FKBP51 protein [120]. The authors were able to confirm *FKBP5* as a target of miR-15a by a luciferase assay and by showing that the seed sequence for this miRNA binding was highly conserved at the 3’UTR of *FKBP5*. Interestingly, this miR-15a-specific downregulation of *FKBP5* expression was later suggested to underlie an important coping mechanism to chronic stress, which warrants further investigation [120].

Finally, by using multiple programs to predict potential miRNA candidates that could target *FKBP5*, three miRNA were identified with potential binding to this gene: miR-142, miR-340, and miR-511 [121]. Although all three miRNAs were shown to regulate *FKBP5* mRNA expression in vitro, only miR-511 was found to robustly reduce FKBP51 protein levels as well [121]. miR-511 has been shown to suppress the glucocorticoid-induced upregulation of FKBP51 in cells and primary neurons, suggesting an important role of this miRNA in FKBP51’s modulatory function in GR activity [122]. In addition, this miR also contributes to age-dependent regulation of *FKBP5* and neuronal development and differentiation [121], suggesting it as a potential target in the modulation of FKBP51. 

### 4.3. Post-Translational Regulation

The final level of regulation comes with the modification of the FKBP51 protein itself by post-translational mechanisms. These include the chemical addition of functional groups or proteins to FKBP51, in addition to the cleavage or degradation of the protein or specific regulatory subunits. In general, the most common post-translational modifications (PTMs) include phosphorylation, glycosylation, ubiquitination, nitrosylation, methylation, acetylation, lipidation, and SUMOylation. 

Overall, evidence for PTMs on FKBP51 is scarce. As opposed to FKBP52, FKBP51’s sequence lacks a phosphorylation site on its FK linker region (a seven- to nine-amino-acid loop that connects the FK1 and FK2 domains) [123]. Although several phosphorylation sites are known for FKBP51 (https://www.phosphosite.org), their functional relevance has not been revealed yet. However, the two peptide modifications ubiquitination and SUMOylation have been reported in the modulation of FKBP51 and its function [124]. SUMOylation of proteins involves the covalent addition of a member of the SUMO (small ubiquitin-like modifier) family of proteins to a protein target. Once bound, the SUMO can have one of three non-mutually exclusive effects: it can interfere with the binding of another protein, it can act as a recruiter of new interacting proteins, or it can lead to conformational changes in the SUMOylated substrate [125]. Ubiquitination also can have a number of different effects, depending on the type of chaining of several ubiquitin moieties, where marking proteins for proteasomal degradation is the prototypic case [126].

FKBP51 can be SUMOylated at Lys^422^ to allow for its association to the GR complex though interaction with Hsp90 [124]. Accordingly, FKBP51 mutants that could not be SUMOylated failed to interact with Hsp90 and GR, suggesting an important modification in FKBP51’s activity on GR signaling [124]. FKBP51 is further modified by the ubiquitin specific peptidase (UPS) 49. Association between USP49 and FKBP51 has been demonstrated, leading to deubiquitination and stabilization of FKBP51 and further consequences with respect to FKBP51-dependent Akt1 activity [127] (Figure 3c).

## 5. Functions of FKBP51

FKBP51 is a protein with many described functions in addition to its GR modulatory properties. Not only does it interact with several other co-chaperones to regulate SRs function, but it has also been suggested to modulate pathways related to the immune function, autophagy, epigenetic remodeling, apoptosis, cell growth, cytoskeleton dynamics, and metabolism, among others. In this section, we will briefly discuss some of its known functions in signaling pathways and their relevance for health and disease.

### 5.1. Regulation of Immune Pathways

The most common role of FKBP51 is linked to its regulatory effect on the action of GR, as previously discussed in this review. Given the known function of glucocorticoids and GR on inflammation [128,129], it is not surprising that FKBP51 has been shown to present direct and indirect effects on immune and inflammatory pathways as well. Specifically, FKBP51 is thought to influence the nuclear factor kappa-light-chain-enhancer of activated B cells (NFκB) system by several different mechanisms. This family of molecules acts as transcription factors that regulate immune and inflammatory responses, among other processes [128], and is considered one of the major regulators of the expression and production of inflammatory cytokines [130].

FKBP51 has been shown to physically associate with the inhibitor of κB kinase α (IKKα), IKKε, TGF-β activated kinase 1, and mitogen-activated protein kinase kinase kinase [131,132], all of which belong to the NFκB signaling pathway. The direct effect of FKBP51 on NFκB, however, is still under investigation. A preclinical study using an animal model of chronic constriction injury showed that FKBP51 knockdown reduced the production of proinflammatory cytokines in the dorsal root ganglion of rats [133], and the same study showed that the inhibition of FKBP51 significantly reduced the activation of NFκB in the same model [133]. One of the mechanisms by which FKBP51 may act here is by inhibiting calcineurin activity [134], which counteracts NFκB activation by dephosphorylating IκB and preventing its proteasomal degradation [131,135]. In addition, a role for FKBP51 in the innate immune response to viral infection has been proposed, which is mediated by the FKBP51 interaction with TNF receptor-associated factor proteins [136]. Finally, FKBP51 has also been shown to modulate the immunosuppressant effects of myeloid-derived suppressor cells by increasing inducible nitric oxide synthase, arginase-1, reactive oxygen species, and enhancing NFκB activity in vivo [137].

### 5.2. Regulation of the AKT Pathway

The AKT/protein kinase B signaling pathway is a pivotal player in the control of cellular function both in health and in pathological conditions [138]. Among several regulatory mechanisms, its activity can be modulated by the phosphorylation status of two sites, namely Ser^473^ and Thr^308^ [139]. FKBP51 has been shown to regulate AKT activity and to function by interfering with its phosphorylation through a scaffolding mechanism. Studies have shown that FKBP51 can interact with AKT at multiple domains independently of their phosphorylation status, either directly or indirectly via Hsp90 [140]. Briefly, FKBP51 can increase the interaction between AKT (which binds to the FK1 domain) and the pleckstrin homology domain leucine-rich repeat protein phosphatase (PHLPP), which binds to FKBP51’s C-terminus [139]. By doing so, FKBP51 facilitates the dephosphorylation of AKT at Ser^473^ by PHLPP. Finally, a recent study also showed that the FKBP51 expression status may determine whether and to which extent antidepressants alter the phosphorylation of AKT Set^473^, which is a key mechanism in the action of these drugs [141].

### 5.3. Regulation of Microtubule Dynamics 

One of the roles proposed for FKBP51 is the regulation of the cytoskeleton, more specifically microtubule dynamics. Studies have shown that it can promote the stabilization of microtubules by interacting with tau in a complex with Hsp90 [142,143]. The mechanism proposed is that phosphorylation of tau leads to its association with FKBP51 and isomerization to a cis configuration; this eventually allows for its dephosphorylation by the phosphatase PP5 and its recycling and stabilization to microtubules [142,143]. Interestingly, as seen in the FKBP51 role on GR modulation, FKBP52 exerts the opposite effect. FKBP52 has been shown to directly interact with tubulin whether polymerized or not, ultimately preventing or inhibiting microtubule formation [144].

Of note, this microtubule-modulating property of FKBP51 and FKBP52 can have important effects on determining neurite length in neuronal cells, which might have further consequences in terms of cognitive and behavioral functions in animals. Accordingly, neurite outgrowth has been shown to reduce after the overexpression of FKBP52 in PC12 cells [144], and the balance between FKBP51 and FKBP52 has been suggested to play a key role during the early mechanisms of neuronal differentiation [145].

### 5.4. Modulation of Autophagy

Based on initial observations that FKBP51 could shift the cellular response to irradiation from apoptosis to autophagy [146], some recent studies have focused on the mechanisms by which FKBP51 could play a role in autophagy and related mechanisms [141,147]. Autophagy is an important and highly regulated cellular process responsible for targeting damaged proteins, lipids, glycogens, and organelles from the cytosol to lysosomes for destruction [148]. The hypothesis that FKBP51 could control autophagy was also supported by evidence of glucocorticoid-induced autophagy [149] and that the autophagy regulators AKT and Beclin1 were among the proteins controlled by Hsp90 and/or its co-chaperone FKBP51 [103].

Accordingly, FKBP51 has been shown to bind to Beclin1 and alter its phosphorylation status, ultimately promoting the induction of autophagic pathways [141]. It does so by interacting with PHLPP and AKT and thereby favoring AKT’s dephosphorylation at Ser^473^, which leads to Beclin1 recruitment and dephosphorylation [141]. Interestingly, antidepressants have been shown to act in a synergistic way with FKBP51 in the regulation of autophagy, and the baseline FKBP51 levels actually correlated with the potential of antidepressants to induce autophagy [141]. Based on recent evidence of an association between autophagy and neurotransmission [150], these FKBP51-mediated processes might eventually underlie pathological mechanisms in depression and other neuropsychiatric conditions, as discussed in Section 5.7.

### 5.5. Regulation of DNA Methylation

Many stress-related conditions and neuropsychiatric disorders show alterations in DNA methylation markers and related mechanisms. Early-life and lifetime stresses have also been consistently shown to induce epigenetic alterations and long-lasting DNA methylation changes, which suggests the GR-FKBP51 system as a player in the establishment and modulation of these processes. In fact, FKBP51 has been recently shown to control the phosphorylation and activity of DNA methyltransferase 1 (DNMT1), a key epigenetic enzyme responsible for the maintenance of DNA methylation [151].

DNMT1 can be regulated by the action of several different molecular interactions and posttranslational modifications [152]. Among its regulatory mechanisms, the phosphorylation of Ser^154^ by cyclin-dependent kinases (CDKs) is of particular importance [153]. An interesting model has been proposed in which FKBP51 and FKBP52 compete for the binding to CDK5 based on their structural similarity [151]. When overexpressed, FKBP51 was shown to displace FKBP52 from CDK5, which decreases the interaction between CDK5 with DNMT1 [151]. This was shown to reduce DNMT1 phosphorylation at Ser^154^ and ultimately its enzymatic activity, leading to a global DNA demethylation in vitro [151]. Altogether, these results suggest a key role for FKBP51 in modulating DNA methylation. As discussed in the previous sections, the *FKBP5* gene itself can also be regulated by methylation, suggesting another modulatory feedback loop with potential impact in health and disease [6]. For example, epigenetic upregulation of *FKBP5* transcription with age has been reported to impair stress-resiliency [117]. This mechanism, together with age-dependent miR-511 activity [121], may also contribute to the genetic association of *FKBP5* with cognitive function in aged individuals [154]. In line with a role in aging, elevated levels of FKBP5 transcripts have been correlated with shorter telomere length in depression [155]. However, it should be noted that FKBP51 has been reported to enhance human telomerase reverse transcriptase activity [156], so that the mechanistic basis requires further investigation.

### 5.6. Regulation of Metabolism

Dysregulation of the HPA axis in obesity has been reported in several studies. Not coincidently, there is emerging literature on the role of FKBP51 in metabolism regulation and obesity-related conditions [157]. In fact, glucocorticoids can induce FKBP5 expression in the adipose tissue, where higher FKBP5 expression has been associated with markers of insulin resistance, such as insulin, homeostatic model assessment of insulin resistance, subcutaneous adipocyte diameter [158], and lower levels of plasma HDL (high-density lipoprotein)-cholesterol [158]. Accordingly, FKBP5 SNPs have been associated with type 2 diabetes [158], insulin resistance, and elevated serum triglycerides [159]. It is likely that the dysregulated HPA axis activity seen in carriers of the *FKBP5* risk variants may mediate lipolysis and insulin resistance, hindering vulnerable subjects that require a rigorous glycemic and lipid control as part of their routine management [159]. In addition, *FKBP5* variants have also been associated with significantly lower weight loss after bariatric surgery [160].

In mice, *Fkbp5* expression was found to be responsive to a high-fat diet and chronic social defeat stress in the hypothalamus and hippocampus, respectively, and hypothalamic *Fkbp5* expression was found to be related to an increased body weight [161]. Work on mice and rats demonstrated that fasting induces FKBP51 in ventromedial, paraventricular, and arcuate hypothalamic nuclei as well [162]. In addition, virus-mediated overexpression of FKBP51 in the hypothalamus led to an elevated body weight in mice on a high-fat diet [162]. Conversely, mice lacking FKBP51 are resistant to diet-induced obesity [163], consistent with the finding that FKBP51 controls adipogenesis [164,165]. Altogether, these findings suggest a novel role of FKBP51 as an important target in metabolic regulation.

### 5.7. Implications in Diseases: Focus on Depression

Because of the plethora of functions and means of regulation of FKBP51, it is not surprising that this system has been associated with several medical conditions. The most common of those are related to acute or chronic exposure to stress, such as most neuropsychiatric disorders, mostly due to FKBP51’s stress inducibility and possibly to its key role in controlling GR function. With different degrees of involvement, FKBP51 has been previously associated with substance abuse [166,167], autism [168], bipolar disorder [169,170], depression [12,116], schizophrenia [171], post-traumatic stress disorder (PTSD) [172,173], suicide [174], attention-deficit/hyperactivity disorder [175], anxiety [176], and the effects of early trauma and stress in general [177,178,179].

Among all of these conditions, depression is the most studied and has been consistently associated with FKBP51 in multiple independent populations. Accordingly, major depressive disorder (MDD) has been linked to a dysfunction in the HPA axis by several studies [180,181,182], and a landmark study from 2004 showed the role of *FKBP5* in antidepressant response causing the initial boom in the study of FKBP51 in clinical populations [12].

A summary of clinical studies investigating the association between depression and FKBP51 is provided in Table 2. As can be seen, the vast majority of studies have focused on associations between depression and the *FKBP5* genotype by investigating multiple single nucleotide polymorphisms (SNPs) throughout the gene (with a particular focus on the initially reported rs1360780). In general, it is currently hypothesized that depressed patients have increased basal FKBP51 levels [155,183,184] (although not observed in all populations [115,185]) that may be leading to a GR resistance [186,187]; alternatively, the basal promoter activity may be less relevant than the stress reactivity of the promoter that is shaped by genotype and epigenotype [6]. Moreover, the *FKBP5* genotype has been reported to interact with the MDD diagnosis to predict structural neuroanatomical changes [116,188,189,190], as well as with prior lifetime trauma and/or stress to increase the risk for depression [191,192,193,194]. A recent meta-analysis performed with 26,582 subjects has also confirmed a significant association between both rs1360780 and rs3800373 with MDD [195].

Finally, 10 studies have investigated the role of *FKBP5* in the response to treatment among depressed patients. As seen in Table 3, eight studies have confirmed this association [12,196,197,198,199,200,201], while two failed to show a role for the *FKBP5* genotype on treatment response [202,203]. A meta-analysis from 2013 was also able to confirm this association and its SNP and ethnicity specificity [204]. The proposed role for FKBP51 in MDD treatment response is also supported by several studies showing the importance and dependence of FKBP51 function in the mechanism of actions of antidepressants, as discussed in Section 5.2, Section 5.4 and Section 6.1 [141,151,205].

## 6. Drugs and FKBP51

FKBP51 is being explored for possibilities for drug development, like other steroid receptor-associated immunophilins [88,227]. In general, two roles for FKBP51 in drug treatment emerge from the literature: (1) FKBP51 organizes signaling pathways that are relevant for the effects of other drugs. Thus, its status needs to be considered when targeting other proteins that link to FKBP51-influenced pathways; (2) FKBP51 may serve as direct drug target. Obviously, targeting FKBP51 directly also has the potential to impact drug effects that use FKBP51-dependent pathways.

### 6.1. FKBP51 Influences the Effects of Other Drugs

The multiple roles of FKBP51 in signaling transduction include pathways that are targeted by drugs. Most notable are anti-cancer drugs and antidepressants. Owing to the observation that the role of FKBP51 may depend on the type of cancer, its contribution to cancer drug treatment cannot be generalized. As reviewed elsewhere, while FKBP51 is upregulated in several human cancers, it is downregulated in others [228]. This may relate to differential importance of growth-regulatory pathways in different cancer types. For example, enhanced expression of FKBP51 can stimulate AR in prostate cancer [229,230] and thus drive malignancy, while as a negative regulator of Akt1 FKBP51 is important for the action of chemotherapeutic agents in pancreatic cancer cells [231].

The dependency of antidepressant drug treatment on the FKBP51 levels has been shown in several studies employing human cell lines, mouse models, and peripheral blood cells from depressed patients and healthy controls [12,141,147,151,205]. More specifically, the effect of antidepressants on mouse behavior and on the GSK3β pathway, global and local (BDNF) DNA methylation, and autophagy depend on FKBP51. Furthermore, the extent of the antidepressant effect on these FKBP51-directed pathways in patient-derived blood cells was predictive of clinical treatment success [141,151,205]. Even though the molecular mechanisms discovered are very plausible, causality has not yet been established.

### 6.2. FKBP51 as A Drug Target

As a member of the immunophilin protein family, FKBP51 binds to the immune suppressive drug FK506 (also called tacrolimus) [232]. FK506 binds to the catalytic pocket thereby blocking the PPIase activity of the FKBPs. Its physiological effects, however, are explained by the fact that it is a large molecule protruding out of the PPIase pocket [233], leading to changes in the interaction profile of the FKBPs. The best investigated example is the FK506-dependent interaction of FKBP12 and other FKBPs with calcineurin, which leads to the inhibition of this phosphatase and thus affects the activity of the transcription factor NFAT (nuclear factor of activated T-cells) as the basis of the immune suppressive effect of FK506 [234]. Since FK506 binds to all members of the FKBP family, other drugs need to be developed to achieve selectivity for FKBP51. It should be mentioned, though, that some of the effects of FK506 have been ascribed specifically to the inhibition of FKBP51, in particular by blocking the AR and thereby inhibiting prostate cancer cell growth [229,230].

Other efforts have been aimed at disrupting the interaction between Hsp90 and TPR proteins, including FKBP51. For example, the coumermycin-A1-related compound novobiocin has been reported as a C-terminal inhibitor of Hsp90 that prevents binding to FKBP51 and other TPR proteins at millimolar concentrations [235]. Furthermore, the small molecule SM145 (which binds between the N and middle domain of Hsp90) reduces interaction between Hsp90 and FKBP51, FKBP52 and other TPR proteins, which also leads to lower levels of FKBP51, FKBP52, and GR [236]. In contrast to N-terminal inhibitors of Hsp90, such as geldanamycin, this C-terminal inhibitor does not mount a heat shock response. Nevertheless, these compounds cannot be considered as specific to FKBP51.

When the structural details of the TPR–Hsp90 interaction were revealed, it was suggested that this interaction might serve as drug target to disrupt the interaction of Hsp90 with TPR proteins, including immunophilins [89,99]. In fact, a hop-derived TPR peptide has been designed, fused to the cell-penetrating antennapedia homeodomain sequence (Antp) [237]. This peptide has been shown to disrupt the interaction of Hsp90 with Hop, but not with PP5 or with FKBP51, and to induce cell death in several cancer cell lines [238,239]. Whether a similar strategy will be successful for targeting the Hsp90-FKBP51 interaction remains an open question. Specificity would be a particular challenge, also when devising EEVD-peptides, and this strategy would be designed to target Hsp90-dependent functions of FKBP51 while leaving others intact. For many of FKBP51’s cellular actions the Hsp90-dependency is not known. In addition, to use GR regulation as an example, it is not known whether FKBP51 exerts a specific action or merely acts by displacing other TPR proteins from Hsp90. In fact, the overexpression of PP5’s TPR domain produced a strong GR-inhibitory effect [81]. Thus, even if inhibitors of FKBP51’s association with Hsp90 could be designed, they probably would act as promoters of some actions of FKBP51, by inhibiting GR on the one hand, and by freeing up FKBP51 from Hsp90 complexes possibly enhancing its Hsp90-independent effects. Nevertheless, very recently, X-ray crystallography and computational modeling have been used for a detailed analysis of the binding of the Hsp90 C-terminal peptide binding to FKBP51 as the basis for a structure-based drug design targeting this interaction [240].

In recent years, ligands for FKBP51 have been synthesized that are selective in the sense that they do not bind to FKBP52 [241,242]. These ligands are often referred to as “inhibitors,” because they block FKBP51’s PPIase activity [241]. However, they may not inhibit all functions of FKBP51 (see above). Nevertheless, these compounds are very promising as they show antidepressant-like, anxiolytic and pain-relieving effects in mouse models [241,243,244]. While these compounds have been carefully designed to bind FKBP51, but not FKBP52, currently it cannot be excluded that binding to other FKBPs contributes to some of their physiological effects. The development of these FKBP51 ligands has greatly benefitted from high resolution crystal structure analyses [245,246]. Crystal structures reveal a particular conformational state, which represents one of many other possible conformations a molecule can typically adopt in solution. Therefore, insights from molecular dynamics simulation, binding free energy calculation, conformational dynamics, and unbinding pathway analysis may contribute to further improvement in the development of these and other FKBP51 ligands [247,248]. In addition, in light of the manifold pathway involvements of FKBP51, it appears useful to determine which of them are actually affected by these drugs, positively or negatively. Whenever possible, rationally designing drugs that affect a subset of FKBP51’s pathway actions is particularly desirable.

## 7. Conclusions

The important role of the FKBP51 system in the regulation of SR activity has led to a series of discoveries in the field of stress-related conditions. Important findings have contributed to the understanding of not only pathophysiological mechanisms of several illnesses (in particular depression and other affective disorders), but also treatment response toward pharmacogenomics approaches and personalized medicine. In parallel, as discussed in Section 6, advancements in chemical and drug design are finally able to pharmacologically target FKBP51 with relatively good precision, with promising opportunities for the treatment of stress-related mental disorders and possibly other diseases.

While this particular focus on FKBP51 research is undeniably important, the preclinical investigation of FKBP51 regulation has led to the discovery of several novel GR-independent functions and signaling pathways involving this protein. Even if they are also somewhat connected to neuronal mechanisms and the response to antidepressants, these novel mechanisms provide the background for innovative investigations of the FKBP51 system in conditions other than the commonly studied neuropsychiatric disorders.

In addition to the growing body of evidence of associations between FKBP51 and medical conditions, it is also noteworthy that this system also seems to influence specific parameters in healthy controls that may or may not have an effect on disease risk. In particular, several studies have reported associations between the *FKBP5* genotype and brain alterations in non-clinical populations. Variants of the rs1360780 have been associated with altered volumes of the dorsal anterior and posterior cingulate cortexes [249], right amygdala [250], left amygdala [251], and the right middle and inferior orbitofrontal regions [250]. Moreover, *FKBP5* polymorphisms have been associated with an altered resting-state activity in the frontotemporal–parietal network in controls [252]. The significance of these findings in increasing the risk for diseases still needs to be investigated, primarily by longitudinal studies.

In summary, accumulating evidence suggests a key role for FKBP51 in multiple cellular functions with potential impact in brain anatomy, metabolism, cognition, risk for diseases, and response to treatments. The multilayered regulation of FKBP51 from its transcription to protein stability provides the basis for the development of innovative molecules exploiting different mechanisms to target FKBP51 and its pathways, with the ultimate goal of improving the lives and prognoses of patients and subjects at risk. To achieve this goal, a more in-depth mechanistic understanding of the multifaceted actions of FKBP51 is required, including the different factors acting synergistically or in an opposing manner.

## Figures and Tables

**Table 1:** Cofactors of Hsp90 and Hsp70 with demonstrated influence on GR activity. For more details, the reader is referred to the original citations provided. (Cofactors of Hsp90 and Hsp70 relevant for GR activity).

Name	Description	Citations
Hsp70 cofactors		
BAG1 (Bcl2 Associated Athanogene 1)	Nucleotide exchange factor for Hsp70 proteins. L- and M-forms inhibit GR in mammalian cells.	[52,54,56,58]
Hip (Hsp70 interacting protein, ST13)	Stabilizes the ADP bound state of Hsp70, opposes the inhibitory effect of BAG1, enhances GR function in the yeast model, possibly also independently of Hsp70.	[59,60,61]
Hsp40 (Heat shock protein 40)	Family of J-domain proteins, enhances Hsp70’s ATP hydrolysis, one of the 5 components in vitro reconstitution system, forms the initial contact in SR folding.	[29,30,62]
Hsp110 (Heat shock protein 110)	Sse1 in yeast, related to and nucleotide exchange factor of Hsp70, enhances GR maturation in the yeast model.	[63,64]
HSPBP1 (Hsp70 binding protein 1)	Nucleotide exchange factor of Hsp70. Overexpression in mammalian cells decreases GR-dependent transcription.	[53,58]
Hsp90 cofactors		
Aha1 (Activator of Hsp 90 ATPase activity 1)	Stimulates the ATPase activity of Hsp90. Enhances GR function in the yeast model and in mammalian cells.	[65,66,67]
Cns1 (cyclophilin seven suppressor)	TPR domain containing protein. Evidence from yeast suggests enhancement of GR function.	[68,69]
Cyp40 (Cyclophilin 40, Cpr6 and Cpr7 in yeast)	TPR domain containing protein and PPIase. Influences GR in some yeast models, no association and effect shown in mammalian cells.	[70,71,72,73]
FKBP51 (FK506 binding protein 51)	TPR domain containing protein and PPIase. Potent inhibitor of GR in mammalian cells. Decreases hormone affinity and delays nuclear translocation.	[7,8]
FKBP52 (FK506 binding protein 52)	TPR domain containing protein and PPIase. Potentiates GR activity in the yeast model. Context-dependency in mammalian cells.	[7,9,74,75]
FKBPL (FK506 binding protein like, WISp39)	TPR domain containing protein with PPIase-like domain. Affects levels, nuclear translocation, and transcriptional activity of GR in a cell line dependent manner.	[76]
P23 (also known as PTGES3, prostaglandin E synthase 3)	Part of the basal in vitro reconstitution system. Promotes Hsp90’s ATP-bound state and substrate-interaction. Context-dependent action on GR activity, chromatin effects.	[2,45,46,47,77,78]
PP5 (protein phosphatase 5, Ppt1 in yeast)	Contains TPR domain protein, dephosphorylates Hsp90. Enhances Hsp90 and GR activity in the yeast model. Overexpression reduces GR activity in mammalian cells.	[71,79,80,81,82]
Tah1 (TPR-containing protein associated with Hsp90)	TPR domain containing protein, in complex with Pih1. Evidence from yeast suggests stimulatory effect on GR.	[27]
SGT1 (Suppressor of G2 allele of Skp1)	Enhances Hsp90 chaperone cycle and GR activity in the yeast model.	[39]
XAP2 (HBV X- Associated Protein 2 = FKBP37)	TPR domain containing protein with PPIase-like domain. Interferes with GR activity upon overexpression in mammalian cells.	[83]
Cofactors for both Hsp70 and Hsp90		
CHIP (Carboxy- terminus of Hsp70- binding protein	TPR domain containing protein, has E3 ubiquitin ligase activity. Reduces GR protein levels and function in mammalian cells.	[71,84]
Hop (Hsp70–Hsp90 organizing protein)	Part of the basal in vitro foldosome reconstitution system. Mediates the transfer from the Hsp70- to the Hsp90-based folding platform.	[2,34,85]
TPR2 (TPR repeat protein 2 = DNAJC7)	Features TPR- and J-domain. Stimulates ATP hydrolysis and polypeptide binding by Hsp70. Excess inhibits GR folding in vitro and activity in vivo.	[71,86]
SGTA (Small Gluta- mine-rich TPR-con- taining Protein α)	TPR domain containing protein. Knock-down and ectopic expression in yeast and mammalian cells evidence an inhibitory role on GR function.	[87]

**Table 2:** Studies investigating the association between FKBP51 and depression.

Ref.	FKBP51 Measure	Sample	Finding
[183]	Protein levels in blood	Patients with MDD (*n* = 30) and healthy controls (*n* = 35)	Higher FKBP51 levels in the cytoplasm of blood cells from depressed patients. Significant correlation between cytoplasmic GR and FKBP5 levels.
[186]	mRNA levels in blood after dexamethasone administration	Two cohorts: (1) *n* = 18 cases/18 controls; (2) *n* = 11 cases/13 controls	Lower *FKBP5* induction after dexamethasone in patients compared to controls.
[185]	mRNA levels in the hippocampus (cornu ammonis and dentate gyrus)	MDD patients (*n* = 43) and healthy controls (*n* = 43)	No difference between groups. No association between *FKBP5* expression and hippocampal volumes.
[206]	mRNA levels in blood	Pregnant women with a lifetime history of mood or anxiety disorders (*n* = 106)	Effect of gestation trimester in upregulating *FKBP5* expression. Reduced magnitude of upregulation of *FKBP5* across pregnancy in depressed women.
[155]	mRNA levels in the hippocampus	MDD patients (*n* = 10) and controls (*n* = 10)	Increased *FKBP5* mRNA levels in patients compared to controls.
[184]	Protein and mRNA levels in the frontal cortex	Controls (*n* = 12), MDD patients (*n* = 12), MDD with psychosis (*n* = 12), HIV^+^ (*n* = 11), and HIV^+^ with MDD (*n* = 11)	Elevated FKBP51 at both the transcript and protein levels correlated with MDD. Higher frequency of the rs3800373 CC genotype in the MDD and MDD/psychosis groups.
[207]	mRNA levels in blood and genotype (rs3800373)	MDD patients at first visit and euthymic at second visit (*n* = 56); euthymic patients at first visit and MDD at second visit (*n* = 30)	Correlation of change in the severity of depressive mood with *FKBP5* gene expression in individuals homozygous for GG of the SNP rs3800373.
[115]	DNA methylation and mRNA levels in PBMCs	Controls (*n* = 20), MDD patients with (*n* = 14) or without serious suicidal ideation (*n* = 10)	Hypermethylation of *FKBP5* gene in MDD patients with concomitant reduced expression of *FKBP5* transcript variants 1, 2, and 3. Further analyses showed that differences were primarily seen in the MDD-suicide group compared to controls.
[116]	Genotype (rs1360780) and DNA methylation	MDD patients (*n* = 114) and healthy controls (*n* = 88)	Interaction between the *FKBP5* genotype and MDD diagnosis on gray matter volumes of several brain regions. Allele-specific positive correlation of the *FKBP5* gene methylation with thickness of the transverse frontopolar gyrus.
[191]	Genotype (rs1360780, rs9470080, rs9394309)	*N* = 1431 participants with available data on early life stress and depressive symptoms at midlife	*FKBP5* SNPs interacted with early life stress exposure, but not with recent stressful life events, in predicting self-reported depressive symptoms in midlife.
[208]	Genotype (rs1360780)	*N* = 131 outpatients with moderate to severe depression	The *FKBP5* rs1360780 T allele was significantly associated with antidepressant treatment increasing suicidal ideation, with a partly drug-specific pattern of association.
[209]	Genotype (rs1360780)	*N* = 922 hospital staff members (depressive state, *n* = 309; non-depressive state, *n* = 613)	Significant association of the *FKBP5* SNP as main effect on depressive state. No significant association between the depressive state and the SNP x stressful life events interaction.
[190]	Genotype (several SNPs)	Inpatients with unipolar depression (*n* = 268) and controls (*n* = 284)	Association between SNPs (especially rs3800373 and rs4713916) in the *FKBP5* gene with vulnerability to unipolar depression. Association of the *FKBP5* genotype with HPA axis activity after citalopram treatment, as well as with right hippocampal volume.
[210]	Genotype, mRNA expression and DNA methylation in blood	Patients with remitted major depression (*n* = 61) and healthy controls (*n* = 55)	Genotype-dependent plasma cortisol response to psychosocial stress exposure in controls (not in patients). rs1360780 T-carrier controls responded with a blunted *FKBP5* mRNA expression after psychosocial stress. Depression- and genotype-specific differences in *FKBP5* methylation in intron 7.
[211]	Genotype (rs1360780)	*N* = 489 children	Significant three-way interaction between *FKBP5* genotype, victimization, and child sex predicting depressive symptoms.
[212]	Genotype (rs1360780)	Children of mothers with (*n* = 81) and without (*n* = 81) a history of depression	Children of depressed mothers who carried the reactive genotype of *FKBP5* rs1360780 exhibited less sustained attention to sad faces and more sustained attention to happy faces (information-processing bias).
[188]	Genotype (rs1360780)	*N* = 115 monozygotic twin pairs discordant or concordant for depression	Additive effect of right hippocampal connectivity alterations and *FKBP5* genotype on depression risk (CC genotype carriers who have low nodal strength in the right hippocampus show higher depression risk).
[213]	Genotype (several SNPs)	Patients with MDD (*n* = 218) and controls (*n* = 742)	Association between five *FKBP5* SNPs (rs1360780, rs9470080, rs4713916, rs9296158, and rs9394309) and MDD. Two haplotype combinations were significantly more frequent in MDD than in controls.
[214]	Genotype (rs1360780)	Patients with a first primary cancer diagnosis (*n* = 7,320)	No association between *FKBP5* genetic variant and use of antidepressants or hospital contact for depression after diagnosis of cancer.
[215]	Genotype (rs1360780, rs9296158, rs3800373, rs9470080)	*N* = 361 pregnant women	No association between *FKBP5* genotypes or haplotypes with depressive symptoms during and after pregnancy.
[187]	Genotype (rs1360780)	Inpatients in a depressive episode (*n* = 68) and healthy controls (*n* = 87)	Significant interaction between MDD and T allele on GR-stimulated *FKBP5* mRNA expression (reduced induction of FKBP5 mRNA in T carriers). GR resistance in T carrier patients.
[216]	Genotype (several SNPs)	*N* = 2928 participants with genetic data and information about depressive symptoms	Significant association between minor alleles of 4 *FKBP5* SNPs (rs9470080, rs9394309, rs7748266, and rs1360780) with decreased cortisol area under the curve and increased risk of depressive symptoms.
[217]	Genotype (rs1360780)	*N* = 344 outpatients with chronic hepatitis C initiating IFN-alpha and ribavirin therapy	No association between *FKBP5* genetic variants and IFN-induced depression.
[192]	Genotype (rs3800373, rs1360780, rs4713916, rs9296158, rs9470080)	*N* = 884 participants (community sample)	No genetic main effect on major depressive episode. Interactions between the five SNPs and traumatic (but not separation) events (especially severe trauma). Homozygosity for the minor allele of selected *FKBP5* SNPs suggested as a risk genotype for the development of a major depressive episode in subjects with prior trauma exposure.
[218]	Genotype (rs1360780, rs4713916, rs3800373)	*N* = 271 children/adolescents whose mothers had experiences at least two major depressive episodes and *N* = 165 controls	No differences in the frequency of the genotypes between the two groups. No association between *FKBP5* genotypes and child and adolescent depression scores.
[12]	Genotype (several SNPs) and protein levels in blood	*N* = 294 depressed individuals and *N* = 339 controls	Individuals with the TT genotype (rs1360780) experienced more than twice as many depressive episodes before the index episode than the other genotypes.
[219]	Genotype (rs1360780, rs3800373, rs9296158, rs9470080)	*N* = 131 adult patients who have received a kidney transplant	*FKBP5* rare alleles at three out of four SNPs in *FKBP5* (rs1360780, rs9296158, and rs9470080) were associated with increased depressive symptoms.
[220]	Genotype (rs3800373)	*N* = 106 school-aged children	Attachment security was negatively associated with depressive symptoms among children with two minor alleles of the *FKBP5* SNP. Maternal attachment anxiety was positively associated with depressive symptoms in these children.
[221]	Genotype (rs1360780)	*N* = 311 physically healthy subjects	Dysfunctional attitudes predisposing to depression were significantly higher in the group with the T-allele than in that without this allele (particularly in achievement and self-control).
[189]	Genotype (rs1360780)	Adult patients with MDD (*n* = 40) and healthy controls (*n* = 20)	T-allele carriers had a later onset of disease compared with CC homozygous patients. Patients expressing the T-allele exhibited functional and structural differences in areas involved in emotional perception and inhibition. Interaction between the risk allele and higher CTQ scores mediates structural alterations.
[222]	Genotype (rs1360780, rs4713916)	Depressed unrelated inpatients with a major depressive episode (*n* = 412) and controls (*n* = 634)	No difference in *FKBP5* genotypes between groups.
[223]	Genotype (rs1360780)	Patients with depression (*n* = 457) and healthy controls (*n* = 2286)	T-allele and TT genotype were overrepresented in depression for men.
[176]	Genotype (rs7757037, rs1360780, rs4713902)	Patients with MDD (*n* = 657) and healthy controls (*n* = 462)	T-allele of rs1360780 was more frequent among patients with MDD with a comorbidity of anxiety disorders, compared to those without.
[193]	Genotype (rs1360780)	*N* = 2157 Caucasian subjects	Significant interaction of physical abuse with the TT genotype of rs1360780 was found to increase depressive symptoms.
[194]	Genotype (rs3800373, rs9296158, rs1360870, rs9470080)	*N* = 236 high-risk, low-income women	*FKBP5* moderation of the indirect effects of maltreatment on depression and dissociation via limbic irritability.
[224]	Genotype (rs1360780)	*N* = 246 Alzheimer’s disease patients with or without major depressive disorder	Significant association of *FKBP5* with MDD in Alzheimer’s disease. C-allele was associated with a higher risk of depression.
[195]	Genotype (rs1360780, rs4713916, rs3800373, rs755658)	Meta-analysis with a total sample size of 26,582, including 14,491 MDD patients and 14,091 controls	No significant association between any of the *FKBP5* SNPs and MDD susceptibility when all samples were pooled. After removing one heterogeneous study, both the rs1360780 T-allele and the rs3800373 C-allele were significantly associated with MDD.
[225]	Genotype (rs1360780, rs3800373, rs9470080)	Meta-analysis with a total sample of 15,109 participants.	Individuals who carry T-allele of rs1360780, C-allele of rs3800373 or T-allele of rs9470080 exposed to early-life trauma had higher risks for depression or PTSD.

GR = glucocorticoid receptor; IFN = interferon; MDD = major depressive disorder; PBMC = peripheral blood mononuclear cells; PTSD = post-traumatic stress disorder; SNP = single nucleotide polymorphism; STAR*D = Sequenced Treatment Alternatives to Relieve Depression.

**Table 3:** Studies investigating the role of FKBP51 in antidepressant response.

Ref.	FKBP5 Measure	Sample	Finding
[196]	mRNA levels in blood	Patients with MDD (*n* = 74) and healthy controls (*n* = 34)	Association between successful antidepressant response and reduction of FKBP5 mRNA levels after 8 weeks of treatment with escitalopram or nortriptyline.
[202]	Genotype (rs1360780, rs3800373)	*N* = 246 geriatric patients with depression	No association between *FKBP5* and clinical outcomes after 8 weeks of treatment with paroxetine and mirtazapine.
[226]	Genotype (rs1360780)	Depressive outpatients (*n* = 159) and controls (*n* = 96)	T allele carriers showed a 2.10 increased risk for non-responding at 4th week to 12-week citalopram treatment (almost significant tendency).
[197]	Genoytpe (rs3800373, rs1360780)	*N* = 304 depressive inpatients with unipolar or bipolar depression	Carriers of the *FKBP5* variants had a trend toward a higher chance to response (mainly in those treated with antidepressant drug combinations or with venlafaxine)
[203]	Genotype (rs1360780)	*N* = 125 outpatients with major depression (*n* = 119) or dysthymic disorders (*n* = 6)	No association between rs1360780 and short-term antidepressant treatment response (fluoxetine; 20 mg/day) or lifetime depressive episodes.
[12]	Genotype (several SNPs)	*N* = 233 depressed inpatients	Association between three SNPs in *FKBP5* (rs1360780, rs1334894 and rs755658) with antidepressant response. In a replication sample, rs1360780 showed a significant association and rs3800373 showed a trend for an association with response to antidepressants.
[198]	Genotype (rs1360780)	*N* = 1953 STAR*D outpatients and *N* = 275 MARS in patients	Interaction between the TT genotype of *FKBP5* rs1360780 and the GG genotype of *GRIK4* (rs12800734) in the prediction of antidepressant response.
[199]	Genotype (rs1360780)	*N* = 298 inpatients with MDD	Interaction between *FKBP5* genotype at rs1360780 and treatment mode. C-allele carriers had a significantly worse outcome when treated naturalistically. TT-genotype subjects showed a superior treatment response across both SSTR and TAU treatment conditions.
[200]	Genotype (several SNPs)	*N* = 529 MDD patients from the Mayo clinic and *n* = 96 from the STAR*D study	Association between the SNP rs352428 with 8-week SSRI treatment response in the Mayo study and 6-week treatment response in the STAR*D replication study.
[201]	Genotype (rs1360780, rs4713916)	Outpatients with non-psychotic MDD (*n* = 1809) and controls (*n* = 729)	rs1360780 was significantly associated with MDD in White non-Hispanics. Significant association between rs4713916 and remission.
[204]	Genotype (rs1360780, rs3800373)	Meta-analysis with a total sample of 2,194 subjects for rs1360780 and 2,049 for rs3800373	rs1360780: In Caucasians T-allele carriers showed a marginal evidence of better response, while in the other/mixed ethnic subgroup a better response was seen in C/C homozygous subjects. rs3800373: In Caucasians C-allele carriers showed a trend of better response.

MARS = Munich Antidepressant Response Signature; MDD = major depressive disorder; SSTR = structured, stepwise treatment algorithm; STAR*D = Sequenced Treatment Alternatives to Relieve Depression; TAU = treatment as usual.

## Abbreviations

BAG1	Bcl2-associated athanogene 1
CDK	cyclin-dependent kinase
DNMT1	DNA methyltransferase 1
FKBP	FK506 binding protein
GR	glucocorticoid receptor
GRE	glucocorticoid response element
HPA	hypothalamus–pituitary–adrenals
HSP	heat shock protein
HSPBP1	Hsp70 binding protein 1
MDD	major depressive disorder
NFκB	nuclear factor kappa-light-chain-enhancer of activated B cells
NR	nuclear receptor
PHLPP	pleckstrin homology domain leucine-rich repeat protein phosphatase
PPIase	peptidylprolylisomerase
PTM	post translational modification
RISC	RNA-induced gene silencing complex
SNP	single nucleotide polymorphisms
SR	steroid receptor
SUMO	small ubiquitin-like modifier
TPR	tetratricopeptide repeat
